# Structural coronary artery remodelling in the rabbit fetus as a result of intrauterine growth restriction

**DOI:** 10.1371/journal.pone.0218192

**Published:** 2019-06-21

**Authors:** Patricia Garcia-Canadilla, Tom de Vries, Anna Gonzalez-Tendero, Anne Bonnin, Eduard Gratacos, Fatima Crispi, Bart Bijnens, Chong Zhang

**Affiliations:** 1 Department of Information and Communication Technologies, Universitat Pompeu Fabra, Barcelona, Spain; 2 Medical Image Analysis, Technische Universiteit Eindhoven, Eindhoven, Netherlands; 3 BCNatal | Fetal Medicine Research Center (Hospital Clínic and Hospital Sant Joan de Déu), University of Barcelona, Barcelona, Spain; 4 Institut d'Investigacions Biomèdiques August Pi i Sunyer (IDIBAPS), Barcelona, Spain; 5 European Synchrotron Radiation Facility, Grenoble, France; 6 Paul Scherrer Institute, Villigen, Switzerland; 7 Centre for Biomedical Research on Rare Diseases (CIBER-ER), Madrid, Spain; 8 Institut de Recerca Sant Joan de Déu, Esplugues de Llobregat, Spain; 9 Institución Catalana de Investigación y Estudios Avanzados (ICREA), Barcelona, Spain; Medicina Fetal México, MEXICO

## Abstract

Intrauterine growth restriction (IUGR) is a fetal condition that affects up to 10% of all pregnancies and is associated with cardiovascular structural and functional remodelling that persists postnatally. Some studies have reported an increase in myocardial coronary blood flow in severe IUGR fetuses which has been directly associated to the dilatation of the coronary arteries. However, a direct measurement of the coronaries’ lumen diameter in IUGR has not been reported before. The aim of this paper is to perform, for the first time, a quantitative analysis of the effects of IUGR in cardiac geometry and coronary vessel size in a well-known rabbit model of IUGR using synchrotron-based X-ray Phase Contrast Tomography Imaging (X-PCI). Eight rabbit fetal hearts were imaged non-destructively with X-PCI. 3D reconstructions of the coronary arterial tree were obtained after semi-automatic image segmentation. Different morphometric features including vessel lumen diameter of the three main coronaries were automatically quantified. IUGR fetuses had more globular hearts and dilated coronary arteries as compared to controls. We have quantitatively shown that IUGR leads to structural coronary vascular tree remodelling and enlargement as an adaptation mechanism in response to an adverse environment of restricted oxygen and nutrients and increased perfusion pressure.

## Introduction

Intrauterine growth restriction (IUGR), linked to placental insufficiency, is one of the leading causes of perinatal mortality and long-term morbidity [1,2] affecting up to 10% of pregnancies. IUGR refers to the condition by which a fetus does not reach their predefined genetic potential weight and is clinically detected prenatally as low estimated fetal weight (under the 10^th^ percentile for gestational age), ideally combined with haemodynamic changes as detected by Doppler Ultrasound, and postnatally confirmed by birth weight [3]. IUGR has been associated with an increased risk of adverse cardiovascular events in adulthood [4]. As a result of oxygen and nutrients restriction, as well as increased placental resistance, IUGR fetuses remodel their circulation and redistribute their cardiac output trying to preserve the oxygen and nutrients supply to the vital organs such as the brain, the heart and the adrenal glands. These adaptive circulatory changes can be observed from Doppler analysis of several vessels including a reduced blood flow in the umbilical artery (UA), caused by the increase in placental resistance, and later, an increased blood flow to the brain as a consequence of the vasodilatation of the cerebral arteries. This phenomenon has been described as the “brain-sparing effect” and has been extensively studied in the last decades. In IUGR fetuses, the increased placental resistance leads to an increase in systemic pressures and thus (bi-) ventricular afterload. With increased IUGR severity, the myocardium is progressively at risk due to the lack of oxygenation and energy supply and the circulation will remodel to compensate where possible. Several studies have reported that fetuses with IUGR therefore show increased coronary blood flow as measured by Doppler ultrasound, which is referred to as the “heart-sparing effect” [5–8]. However, circulatory and cardiac remodelling can’t fully compensate for the hypoxia and lack of nutrition [9,10].

Compared to other larger organs, the heart has the highest oxygen consumption and hence coronary arterial flow is regulated in order to maintain an adequate supply of oxygen and nutrients to the myocardium. Under acute hypoxemia, the coronary flow, through vasodilatation, can dynamically increase up to five-fold as measured in human fetuses [5] as well as in a sheep fetal model of IUGR [11]. Although the acute increase in coronary flow can be beneficial during prenatal life, when there is a chronic need for increased flow, this can lead to an altered development of the cardiovascular system that persists postnatally. For example, recent studies in humans have shown that IUGR fetuses have more globular and less efficient hearts and these changes are still present during childhood [12]. A study performed in a fetal sheep model showed that IUGR alters mechanical wall properties and impairs cardiomyocyte maturation [13]. However, the impact of the remodelling of the coronary vascular tree on human fetuses and the long-term consequences remain unknown.

Acute coronary vasodilatation is indirectly assessed by measuring coronary blood flow with Doppler ultrasound [5,6] since, with vasodilatation, the maximal blood flow will increase. However, given the size of fetal coronary arteries, the measurement of coronary arterial blood flow is challenging and therefore the estimation of vessel diameter changes by Doppler ultrasound is not accurate. Thus, a more direct measurement of the vessel size is needed to quantitatively assess structural coronary remodelling in IUGR. This limitation can be overcome by the use of experimental animal models of IUGR together with high resolution imaging techniques. We have recently shown that synchrotron-based X-ray Phase-Contrast tomography Imaging (X-PCI) is a novel high-resolution technique that allows the visualisation and quantification of the microstructure of whole hearts in great detail, in 3D and non-destructively [14]. As far as we are aware of, there is no study quantitatively measuring the coronary vessels in IUGR fetuses.

The aim of this study was to perform a high-resolution quantitative analysis of the coronary arteries by means of synchrotron-based X-PCI to assess and quantify the remodelling of the coronary arteries in fetuses of a validated IUGR animal model, which has previously been shown to reproduce biometric and hemodynamic changes of human IUGR. Among the different existing animal models of IUGR, the one used in the present study is based on the selective ligature of uteroplacental vessels in the pregnant rabbit, thus combining restriction of nutrients and oxygen, which mimics severe IUGR due to placental insufficiency in humans [15,16]. In particular, we looked at changes in cardiac shape, left ventricular mass (LVM), vessel lumen diameter and vessel volume of the fetal rabbit heart.

## Materials and methods

Animal handling and all procedures were performed in accordance with the regulations and guidelines and with the approval of the Animal Experimental Ethics Committee of the University of Barcelona (permit numbers: 313/11 with date of approval 19 July 2011). New Zealand white rabbits were provided by a certified breeder (Granja San Bernardo, Navarra, Spain) and dams were housed for 1 week before surgery in separate cages on a reversed 12/12 hour light cycle, and fed with standard diet and water ad libitum. Three different dams were used to reproduce a validated IUGR rabbit model following the method previously described [17]. Briefly, at 25 days gestation, tocolysis (progesterone 0.9 mg/kg, intramuscularly) and antibiotic prophylaxis (Penicillin G 300.000 UI, intravenously) were administered before surgery. Ketamine (35 mg/kg) and xylazine (5 mg/ kg) were given intramuscularly for anaesthesia induction. Both uterine horns were exteriorised and one was selected as IUGR, in which selective ligature of 40–50% of utero-placental vessels of each gestational sac was performed. No additional procedure was performed in the horn assigned as control. The abdomen was closed afterwards, and animals received buprenorphine (0.4 mg/kg/24 h, subcutaneously) for 48h, as postoperative analgesia and were kept in regular conditions and fed a diet of standard chow and water ad libitum. Five days after surgery, at 30 days gestation, a caesarean section was performed under the same anaesthetic procedure as described above. All living fetuses were identified and weighted. After anaesthesia with intramuscular ketamine(35 mg/kg) and xylazine (5 mg/kg), the fetal chest was opened. Heparin 500U was administered as well as saturated potassium chloride to arrest the heartAfter thoracotomy, a phosphate-buffer saline solution was used to rinse and 10% formalin to fix the heart, which was excised and immersed in formalin. The mothers were sacrificed during anaesthesia, with pentobarbital (200mg/kg). Before imaging, hearts were dehydrated with ethanol and immobilised in 1% agarose to avoid motion artefacts. A total of 8 samples, 4 controls and 4 IUGR were used. Samples were named CTRL1-CTRL4 and IUGR1-IUGR4, where the first four correspond to controls and the last four to IURG fetal hearts.

### Image acquisition

Whole heart image acquisition was performed at the European Synchrotron Radiation Facility (ESRF—ID19 beamline, Grenoble, France) by means of propagation-based X-ray Phase contrast tomography (X-PCI) following the same procedure described in Gonzalez-Tendero et al [14]. Briefly, the image acquisition was done using a 19keV parallel X-ray beam. The sample was kept at room temperature and placed at the centre of the rotator stage located at a distance of 110cm from the detector (FReLON CCD). The field of view was 5.68 x 15.96 mm with isotropic pixel size of 7.43μm. The sample was rotated over 360° acquiring 2499 projections (exposure time = 0.3s). Since the hearts were larger than the field of view, from four to five sequential acquisitions, from base to apex, were necessary to cover the whole heart along its long axis. Additionally, 41 flat images and 21 dark images were acquired in order to apply the flat-field correction before the reconstruction procedure. Therefore, total acquisition time was approximately 1–1.25h/sample. Then, each set of projections was reconstructed using filtered back projection [18]. Reconstructed volumes were then converted to 16-bit tiff and stitched together in order to obtain a single data set for each sample.

### Biometric cardiac measurements

In order to estimate the LVM we used the truncated Ellipsoid Method [19], as it is one of the most commonly used methods in 3D echocardiography. Therefore, the LVM was estimated as follows:

$$LVM=1.05\left\{{\left(b+t\right)}^{2}\left[\frac{2}{3}(a+t)+d-\frac{d^{3}}{3{\left(a+t\right)}^{2}}\right]-b^{2}\left[\frac{2}{3}a+d-\frac{d^{3}}{3a^{2}}\right]\right\}$$ (1.1)

where *a* is the long or semi-major axis extending from the widest left ventricle (LV) short-axis to the LV apex, *b* is the widest LV short-axis radius, *d* is the truncated LV long-axis extending from the widest short-axis to the mitral anulus place and *t* is the mean LV wall thickness derived from the short-axis plane. Finally, 2D LV sphericity index was calculated as base-to-apex length divided by the basal short-axis diameter.

### Segmentation of the coronary arteries

In order to reduce illumination artefacts and homogenise the image contrast, local contrast was normalised by means of the build-in function *Normalize Local Contrast* in *Fiji* [20]. More details about the pre-processing steps are provided in the Supplementary material.

The coronary arterial tree was semi-automatically segmented with *Carving* module from the open-source image analysis software *ilastik* [21]. The *Carving* module uses a seeded watershed algorithm for interactive object carving from image data [22]. For each object of interest, in our case the coronary vascular tree, the algorithm requires that the user provides ‘inside’ and ‘outside’ seeds as input. From these seeds, an initial segmentation is automatically calculated using a biased watershed algorithm that can be refined interactively. The seeded watershed relied on discernible object boundaries in the image data. A step filter was used to create the boundary map. Details about all the steps of the segmentation of the coronary arteries are detailed in the Supplementary material and illustrated also in an online video [23].

### Quantification of vessel lumen diameter

Once the whole coronary arterial tree was segmented, the local vessel lumen diameter was approximated as the diameter of the largest sphere that fits within the structure using *Fiji*’s plugin *BoneJ* [24]. Then, the 3D skeleton was computed in order to find the vessel centrelines using *Fiji*’s plugin *Skeletonize 3D* [25], which erodes the objects’ surface iteratively until only a 1-pixel wide skeleton is obtained. Erosion was performed symmetrically in order to guarantee the medial position of the skeleton lines and such that the connectedness of the objects was preserved. To compute the vessel’s volume the aortic root was first manually removed from all the segmented images.

Finally, the vessel lumen diameter profiles were obtained for the right coronary artery (RCA), left coronary circumflex artery–including the left main stem (LCX), and left anterior descending (LAD) artery. To do that, a Dijkstra shortest path algorithm [26] implemented in *MeVisLab* [27] was used to find the shortest path between a start and an end point within the 3D skeleton calculated previously for each of the three arteries (see online video [28]). The lumen diameter of the RCA, LCX and LAD were computed for all the 8 samples. Then, in order to compare the lumen diameter profiles of the different arteries among all the subjects, they were normalised according to their corresponding LVM using the following equation:

$$z_{i}=\frac{x_{i}-\min(x)}{\max(x)-\min(x)}$$ (1.2)

where *z*_*i*_ is the normalised lumen diameter profile, *x*_*i*_ is the lumen diameter profile divided by the LVM and min(x) and max(x) are the minimum and maximum values of the lumen diameter divided by the LVM for the same vessel respectively.

Finally, a linear fitting, *y = β*_*1*_*x+ β*_*0*_, was done to all the normalised lumen diameter profiles and their linearity coefficient (*R*^*2*^) were also calculated.

### Statistics

For biometric, vessel lumen diameter and vessel volume data, normality was assessed by the Lilliefors test based on Kolmogorov–Smirnov test. Normal-distributed quantitative variables were analysed by Student’s t-test. Non-normal distributed variables were analysed with the non-parametric Mann–Whitney U test. Differences were considered significant with probability values of p < 0.05. The statistical analysis was performed in SPSS® (IBM Corp. Released 2016. IBM SPSS Statistics for Macintosh, Version 24.0. Armonk, NY, IBM Corp) and in Matlab 2018a (The Mathworks Inc, Natick, MA, USA).

## Results

Morphometric parameters for all the 8 fetal rabbit hearts are summarised in Table 1. Absolute fetal body weight was significantly lower in IUGR as compared to controls. While there is a trend towards a decrease of LVM in IUGR compared to controls (*p* value = 0.061), the LVM to body weight ratio was similar among groups. As compared to controls IUGR fetuses had more globular hearts (lower sphericity index). Moreover, when LV wall thickness was normalised to LVM, IUGR fetuses showed increased normalised wall thickness compared to controls suggesting that IUGR fetuses have hypertrophic hearts although this difference was not statistically significant (6.88 ± 0.54 vs. 5.19 ± 0.75, *p* value = 0.061 calculated with Student’s t-test).

**Table 1:** Morphometric parameters in IUGR and control fetuses.

	Controls (n = 4)	IUGR (n = 4)	*p* value
**Body weight (g)**	49.15 ± 7.51	31.38 ± 10.15	0.0054
**Base-to-apex length (mm)**	8.49 ± 0.53	7.08 ± 0.73	0.1093
**Basal diameter (mm)**	6.49 ± 0.17	6.59 ± 0.25	0.6471
**LV Sphericity index**	1.31 ± 0.05	1.07 ± 0.08	0.0378
***Left ventricular mass (LVM) estimation***			
**a (mm)**	4.77 ± 0.42	4.44 ± 0.53	0.3830
**d (mm)**	3.53 ± 0.45	2.82 ± 0.13	0.0495
**b (mm)**	2.51 ± 0.17	2.32 ± 0.04	0.1551
**t (mm)**	1.76 ± 0.16	1.26 ± 0.20	0.0617
**LVM (g)**	0.35 ± 0.08	0.19 ± 0.04	0.0609
**LVM/body weight x 100**	0.70 ± 0.06	0.65 ± 0.27	0.7820
**Vessels volume (mm**^**3**^)*	2.17 ± 0.57	2.43 ± 0.41	0.6857

Data shown as mean ± SD for the normally distributed variables and as median ± mean absolute deviation for the non-normally distributed variables (indicated by *). *a*, *d*, *b* and *t* are the parameters from Eq 1.1 to compute the left ventricular mass (LVM), which correspond to: *a* is the long or semi-major axis extending from the widest left ventricle (LV) short-axis to the LV apex; *b* is the widest LV short-axis radius; *d* is the truncated LV long-axis from extending from the widest short-axis to the mitral anulus place; *t* is the mean LV wall thickness.; g: grams; mm: millimetres; *p* value was calculated with Student’s t-test for the normally distributed variables or with Mann-Whitney U test for the non-normally distributed variables.

**Fig 1:**
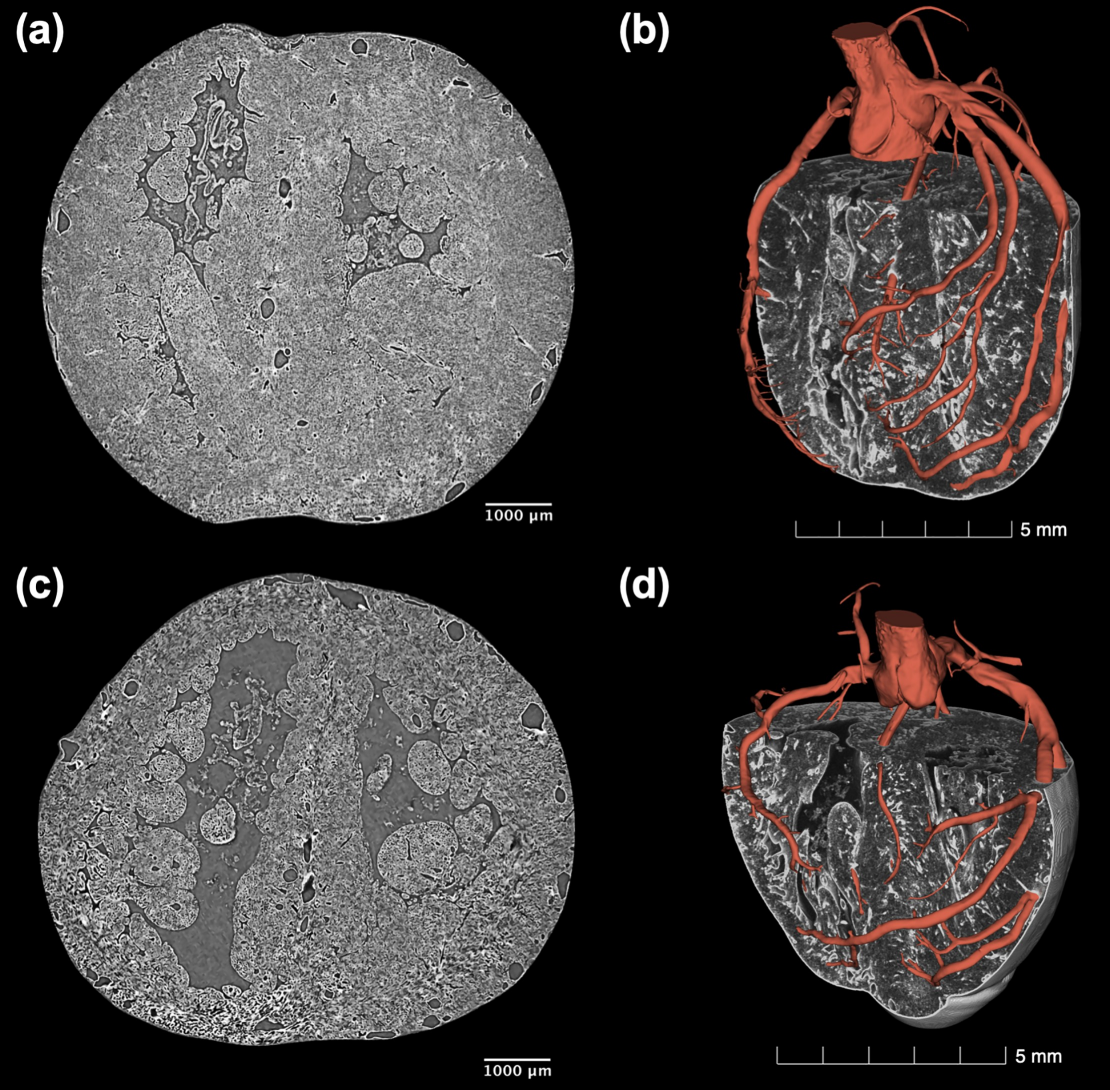
Volumetric visualization of the cardiac anatomy and coronary tree of one control and one IUGR fetal rabbit heart. Short-axis and volume rendered images together with the three-dimensional reconstruction of the coronary arterial tree in red of (**a**-**b**) one control (CTRL1) and (**c-d**) one IUGR (IUGR3) fetal hearts showing clearly that IUGR heart is smaller with dilated coronaries.

Fig 1 shows short-axis cuts of volume rendered images of one control fetal heart (CTRL1) and one IUGR fetal heart (IUGR3), together with their 3D segmented arterial trees. The IUGR heart is clearly smaller than the control heart showing also dilated coronary arteries.

**Fig 2:**
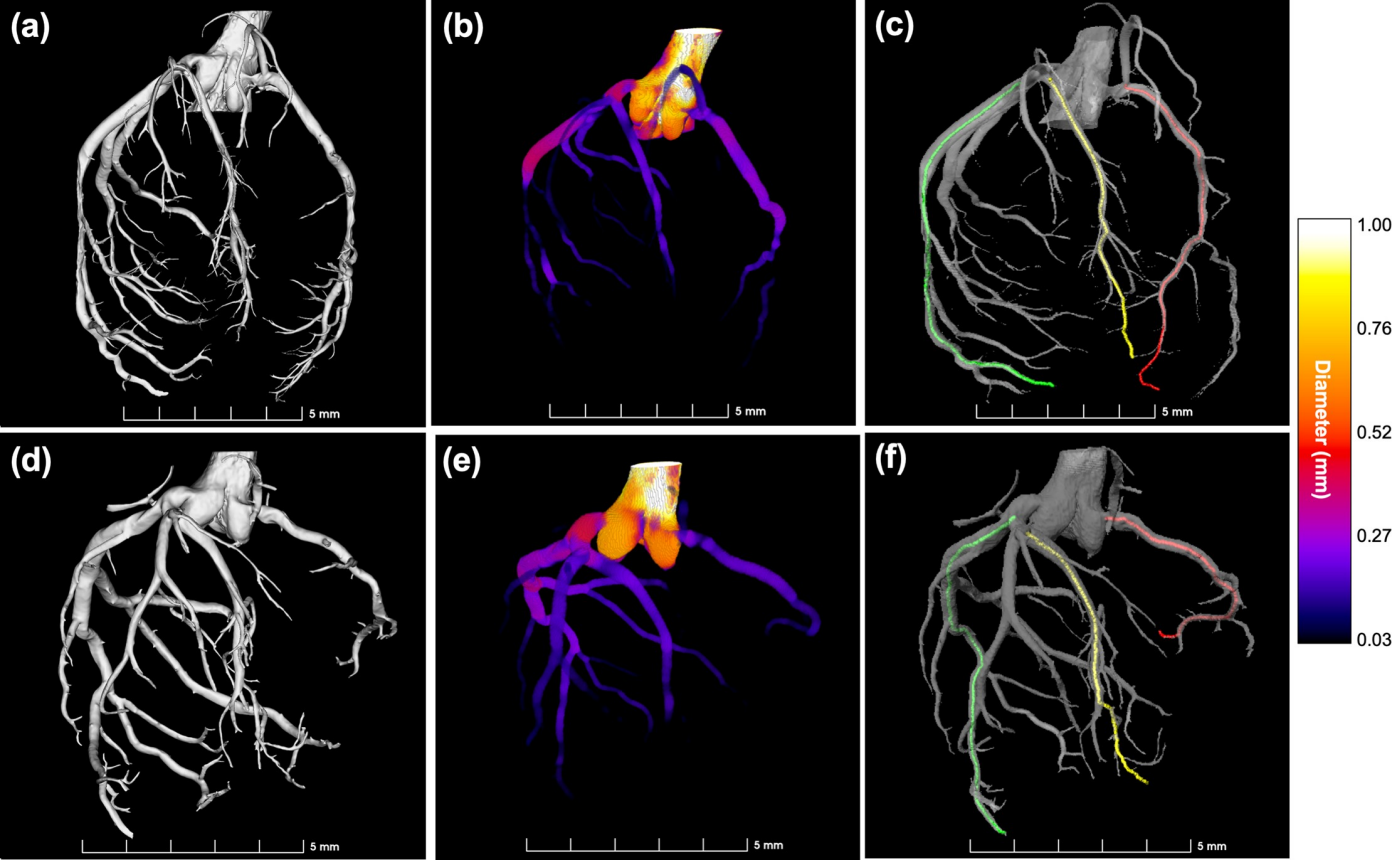
Segmentation of the coronary arterial tree and subsequent estimation of the vessel lumen diameter in one control and one IUGR fetal rabbit heart. Three-dimensional representation of the segmented coronary arterial tree for (**a**) control (CTRL1) and (**d**) IUGR (IUGR3) fetal hearts together with their corresponding colour visualization of the estimated vessel lumen diameter (**b**) and (**e**). The three main coronaries that were quantified in each dataset are indicated in different colours: right coronary artery (RCA) in red, left coronary circumflex artery–including the left main stem (LCX) in green, and left anterior descending (LAD) artery in yellow for the control (**c**) and the IUGR (**f**) fetal hearts.

Fig 2 shows an illustrative example of the segmented 3D coronary arterial tree for one control (CTRL1) and one IUGR (IUGR3) fetal rabbit hearts (Fig 2(A) and 2(D)) as well as a colour visualization of the estimated vessel lumen diameter (Fig 2(B) and 2(E)) showing the coronary dilatation in the IUGR fetal heart. The rest of cases are shown in S4 Fig.

**Fig 3:**
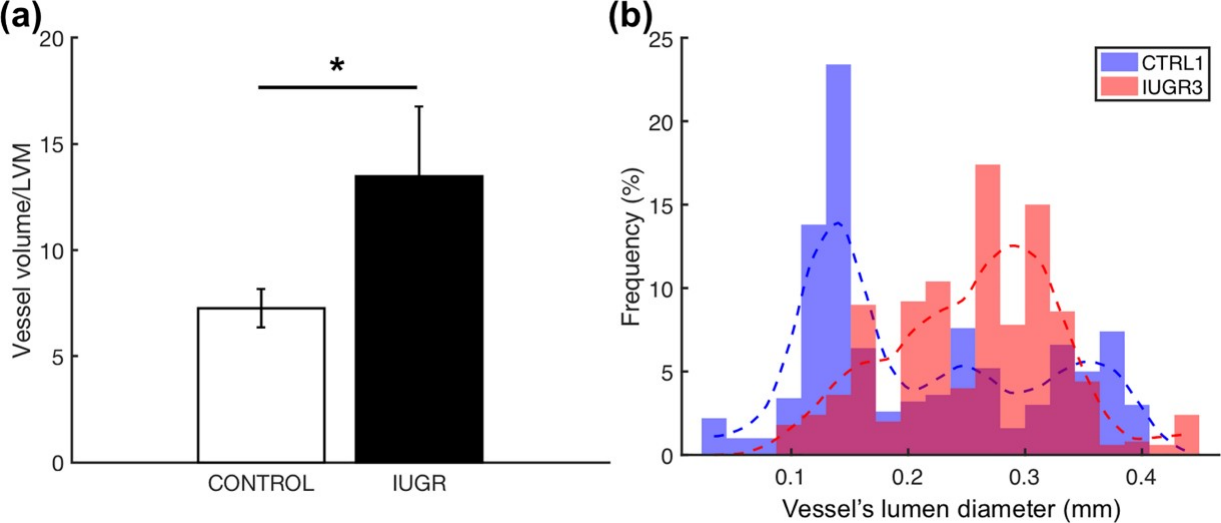
Quantification of the coronary arterial tree size in the control and IUGR groups. (**a**) Ratio between vessel’s volume and left ventricular mass (LVM) (*p* value = 0.0209 calculated by Mann-Whitney U test). (**b**) Histograms of the vessel lumen diameter of all three main coronary arteries: right coronary artery (RCA), left coronary circumflex artery–including the left main stem (LCX) and left anterior descending (LAD) for one control (CTRL1) (blue) and one IUGR (IUGR 3) (red) fetal hearts.

The vessel volume to LVM ratio was significantly increased in IUGR fetuses compared to controls (13.47 ± 3.29 vs. 7.25 ± 0.89, p = 0.0209 calculated with Mann–Whitney U test) thus demonstrating also the coronary arteries dilatation (Fig 3(A)). Moreover, the histograms of all three (RCA, LCX and LAD) vessel lumen diameters for CTRL1 and IUGR3 show that the distribution of coronary size is shifted to the right in the IUGR fetus (Fig 3(B)).

**Fig 4:**
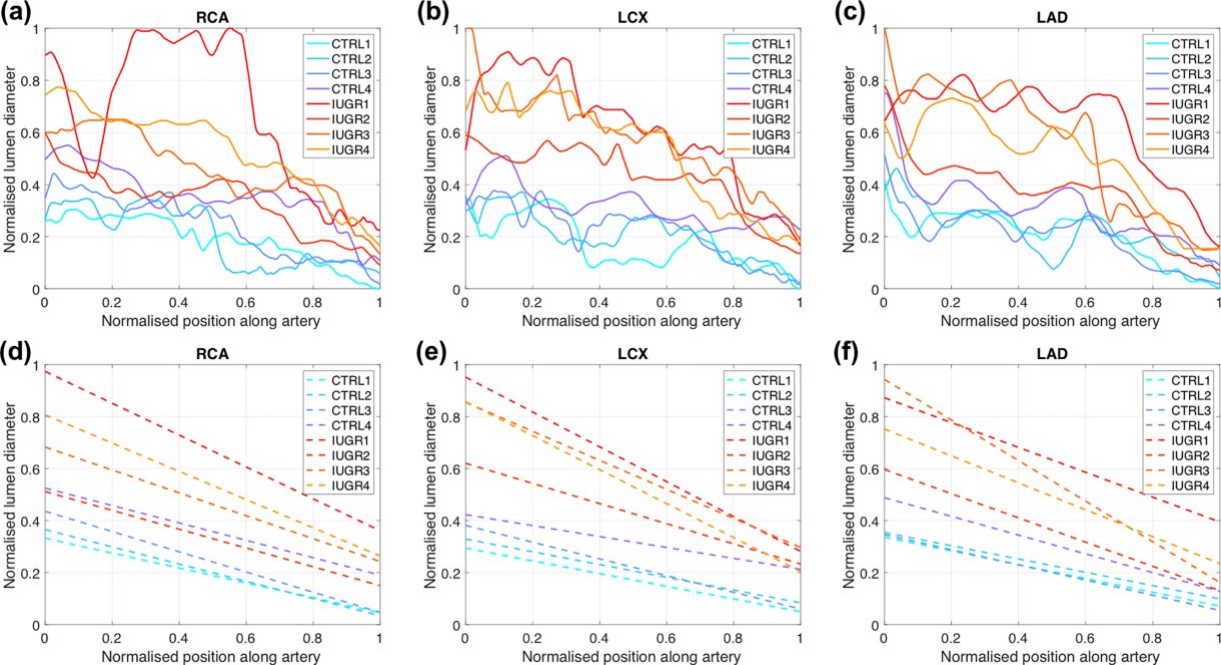
Lumen diameter quantification of the three main coronary arteries shows that IUGR fetuses have dilated coronary arteries compared to controls. Normalised vessel lumen diameter (solid lines) of the (**a**) right coronary artery (RCA), (**b**) left coronary circumflex artery–including the left main stem (LCX) and (**c**) left anterior descending (LAD) artery and their corresponding linear fitting (dashed lines) (**d-f**) for the four controls (CTRL1-CTRL4) and the four IUGR (IUGR1-IUGR4) fetuses. Controls were plotted in blue colours while IUGR were plotted in red colours.

Fig 4 shows the plots of the vessel lumen diameter profiles for the three main coronary arteries: RCA, LCX and LDA, and for all the eight fetuses (Fig 4(A)–4(C)) together with their corresponding linear fitting (Fig 4(D)–4(F)). All four IUGR fetuses showed dilated coronaries relative to their LVM as demonstrated by the steeper and shifted toward higher values of lumen diameter profiles as compared to controls. Table 2 shows the parameters of the linear fitting (*β*_*1*_, *β*_*0*_ and *R*^*2*^) for both control and IUGR groups, where both *β*_*1*_ (slope) and *β*_*0*_ (intercept) parameters of LCX and LAD were significantly different between groups, thus demonstrating also that IUGR shows dilated coronary arteries compared to controls. These differences were more significant in LCX than LAD and RCA.

**Table 2:** Results of the linear fitting *y = β*_*1·*_*x+ β*_*0*_, for the lumen diameter profiles of right coronary artery (RCA), Left Circumflex artery–including left main stem (LCX) and left anterior descending artery (LAD).

	Controls (n = 4)	IUGR (n = 4)	*p* value
***Right Coronary artery (RCA)***			
**β**_**1**_	-0.34 ± 0.04	-0.49 ± 0.11	0.1143
**β**_**0**_	0.41 ± 0.09	0.74 ± 0.20	0.0555
**R**^**2**^	0.82 ± 0.09	0.75 ± 0.24	0.6283
***Left Circumflex artery–including left main stem (LCX)***			
**β**_**1**_	-0.25 ± 0.05	-0.56 ± 0.13	0.0239
**β**_**0**_	0.35 ± 0.06	0.82 ± 0.14	0.0086
**R**^**2**^	0.66 ± 0.10	0.84 ± 0.05	0.0277
***Left Anterior Descending artery (LAD)***			
**β**_**1**_	-0.29 ± 0.05	-0.56 ± 0.15	0.0378
**β**_0_*	0.35 ± 0.05	0.81 ± 0.12	0.0286
**R**^2^*	0.71 ± 0.03	0.77 ± 0.09	0.3429

Data shown as mean ± SD for the normally distributed variables and as median ± mean absolute deviation for the non-normally distributed variables (indicated by *). β_1_ corresponds to the slope of the linear fitting;, β_0_ corresponds to the intercept of the linear fitting; R^2^ corresponds to the linearity coefficient of the linear fitting. *p* value was calculated with Student’s t-test for the normally distributed variables or with Mann-Whitney U test for the non-normally distributed variables.

## Discussion

In the present study we have quantitatively shown that IUGR fetuses showed increased coronary lumen diameter as compared to controls, thus demonstrating the dilatation of the coronary vascular tree occurring in IUGR as an adaptation mechanism in response to an adverse environment of restricted oxygen and nutrients and increased perfusion pressure.

To our knowledge this is the first study which performs a detailed quantitative analysis of the coronary vascular remodelling in an animal model of IUGR. The present study provides evidence of cardiac and vascular remodelling in an experimental model of IUGR which has smaller and more globular hearts as well as dilated coronary arteries, similarly to what is observed in human growth restricted fetuses [12,29]. As described by Crispi et al, the heart becomes more globular to maintain stroke volume with less contraction force and also reducing local wall stress to better tolerate pressure overload occurring in IUGR. Regarding the vasodilatation of the coronary arteries, our findings are also consistent with both human [29] and experimental studies which have reported increased myocardial blood flow in IUGR fetuses [11,13]. However, in all these studies coronary vasodilatation was indirectly assessed by measuring Doppler velocities in coronaries, as opposed to direct quantification of vessel lumen diameter. We have recently demonstrated that X-PCI is a novel high-resolution technique that allows a detailed post-mortem visualisation and analysis of the overall cardiac geometry as well as the different macro- and micro-structures such as valves, trabeculations, false tendons, coronary vasculature, etc. in 3D and non-destructively, thus allowing the quantification of cardiac remodelling from cell to organ levels [14]. However, in the current study a quantitative evaluation of the coronary arterial size was performed for the first time in the setting of IUGR using X-PCI images of fetal rabbit hearts, thus demonstrating that IUGR fetuses have dilated coronaries as compared to controls.

The coronary circulation is an oxygen-sensitive vascular bed and the regulation of myocardial perfusion is critical for optimal cardiac function. Long-term regulation of coronary blood flow involves vascular remodelling while short-term regulation is achieved through autoregulation and changes in perfusion pressure [29]. The persistent changes of coronary vasculature in IUGR are due to the hemodynamic redistribution occurring as a consequence of the deterioration of placental function and resistance, aiming to maintain an adequate oxygen level to vital organs including the brain, the heart and the adrenal glands under the adverse environment of oxygen and nutrient restriction. The first event that can be detected antenatally is the redistribution of flow towards the brain and is known as “brain-sparing effect” [30,31] which can be diagnosed from an increased blood flow in the middle cerebral artery and reduced or reversed diastolic flow in the aortic isthmus. During further deterioration, coronary perfusion is also increased to maintain a high supply of oxygen to the myocardium which is known as the “heart-sparing effect” [5–8]. To increase coronary flow, either the mechanism of acute vasodilatation can be employed, leading to an increased blood velocity in the vessel, or the arteries can structurally remodel by increasing their size and maintaining baseline flow velocities. These complementary adaptations can be difficult to detect with ultrasound imaging and given that early impairment may be subtle. Only when the degree of impairment is high or acute, an increase in both systolic and diastolic velocities in the coronaries can be observed [32].

We have shown that the three main coronary arteries, LCX, RCA and LAD, are dilated in IUGR fetuses as compared to controls but the biggest difference is seen in the LCX. The reason for that can be explained by the fact that LCX is the artery that supplies more blood to the myocardium as compared to RCA and LAD. About a 80% of the total coronary blood flow is supplied by left coronary artery [33] and it is distributed to the left side of the heart: left atrium, left ventricle and interventricular septum. However, LCX flow in human depends on coronary artery dominance [34] and this might vary by species. Nevertheless, there is no information for the particular case of rabbits. Recent studies have also shown that the number of cardiomyocytes was reduced in both ventricles in response to IUGR [35–37] and this persists until a year after birth [38], while hypertrophic remodelling of cardiomyocytes and a decrease in the number and length of capillaries was only observed in the LV [37]. Another study in lambs has also reported that the length of capillaries in the LV was decreased in those with low birthweight [39]. The authors hypothesised that the reduction of the number of cardiomyocytes in both ventricles can be explained by the change in volume occupied by the vessels compared to myocytes and interstitial space due to the dilatation of coronary arteries [37]. However, it seems that these changes in the main coronaries are not accompanied by changes in the same direction in the microvasculature. Further experiments are warranted to investigate these unequal structural changes at the microstructural level between both ventricles in IUGR fetus.

While the coronary vascular tree in the developing heart is very plastic and has the ability to remodel to meet oxygen demands, this remodelling might be detrimental postnatally and towards adulthood and might be a risk factor resulting in increased vulnerability to cardiovascular disease. In the present study, we have quantitatively demonstrated that IUGR fetuses of a validated animal model of severe IUGR have dilated coronary arteries compared to controls. Whether similar findings would be present in moderate (late onset) IUGR remains to be studied. Additionally, whether this vascular remodelling persists postnatally, as well as its long-term consequences remain unknown. In this regard, it was described that children at the age of 9, but born with low birthweight, had relatively smaller total coronary arteries as well as smaller outflow tracts and aortic roots [40]. However, whether this was associated with vessel wall alterations is unknown nor is it clear if these were IUGR cases or what the sizes of the cardiac structures were at birth. Therefore, further research is needed to elucidate the expected vascular remodelling in all etiologies leading to low birthweight and their relation to long-term consequences. In our animal model, the long-term follow up is very challenging due to the high perinatal and postnatal mortality, which limits survival to only the milder IUGR cases [16], while in humans, in-vivo fetal or neonatal assessment of the coronaries is currently not feasible.

It has been described, both in human and in different animal models of IUGR, that males are more sensitive to insults during development and females are protected against development of adult disease in response to fetal insults [41,42]. While sex thus might be of importance when studying fetal remodelling, we acknowledge that one of the limitations of our study is that sex of the animals could not easily be determined at the time of birth in fetal rabbits. Therefore, it was not possible to analyse differences in vessels, heart size and birth weight by gender. Future studies are warranted to assess differences in cardiovascular remodelling by gender. Another limitation of the present study is that we have assumed a linear lumen diameter-length relationship for simplicity while coronary vasculature obeys Murray’s which states that the cube of the radius of a parent vessel equals the sum of the cubes of the radii of the daughters [43]. Finally, our measurements where performed ex-vivo in a relaxed state in which the coronary arteries were perfused and fixed and therefore the exact amplitudes of the measurements can be different compared to in-vivo as has been previously reported [44]. However, this would unlikely change the comparison of the groups reported in our study.

## Conclusions

In conclusion, we have quantitatively demonstrated that IUGR leads to a persistent dilatation of the coronary arteries in an animal model of IUGR by means of the novel synchrotron X-PCI technique. However, the long-term consequences of the coronary vascular remodelling are still unknown. Therefore, future studies are warranted to assess the potential long-term persistence of these structural changes and to evaluate the association of fetal cardiac dysfunction and increased risk of cardiovascular disease in IUGR.

## Supporting information

S1 Fig Illustration of the illumination artefacts in one of the datasets and how these artefacts are reduced by the normalisation of local contrast. A longitudinal reslice of the image data set corresponding to sample IUGR4 (**a**) before and (**b**) after applying the Normalize Local Contrast filter where a lot of illumination artefacts can be clearly seen in the original dataset.
(TIFF)

S2 Fig Illustration of an incomplete segmentation of the coronary arteries in one dataset due to the illumination artefacts. (**a**) Incomplete segmentation of the coronary arteries (in green) when illumination artefacts are present (dashed line white box). **(b**) Three-dimensional reconstruction of the partial segmentation of the coronary arterial tree.
(TIFF)

S3 Fig Comparison between bright lines and step edges filters performance in small vessels. Results of the image filtering with (**a**) bright lines and (**b**) step edges filters to generate boundary maps in the same region. It can be clearly seen that the lumen of small vessels (red boxes) is only distinguishable in the step edge filtered image.
(TIFF)

S4 Fig Segmentation of the coronary arterial tree and subsequent quantification of the vessel lumen diameter in three controls and three IUGR fetal rabbit hearts. Three-dimensional representation of the segmented coronary tree (left panel) together with their corresponding colour visualisation of the estimated lumen diameter (middle panel) for the reaming 3 control (**a-c**) and 3 IUGR (**d-f**) fetal hearts. The right panel depicted the three main coronaries that were quantified for each dataset indicated in different colours: right coronary artery (RCA) in red, left coronary circumflex artery–including the left main stem (LCX) in green, and left anterior descending (LAD) artery in yellow.
(TIFF)

S1 File Supplementary methods. Supplementary methods.
(PDF)

S2 File NC3Rs ARRIVE guidelines checklist file. Completed ARRIVE Guidelines Checklist file.
(PDF)
