# Supplementary figures and images for: Structural coronary artery remodelling in the rabbit fetus as a result of intrauterine growth restriction
Source: PLoS One. 2019 Jun 21;14(6):e0218192. doi: 10.1371/journal.pone.0218192 (PMC6588274; doi:10.1371/journal.pone.0218192)

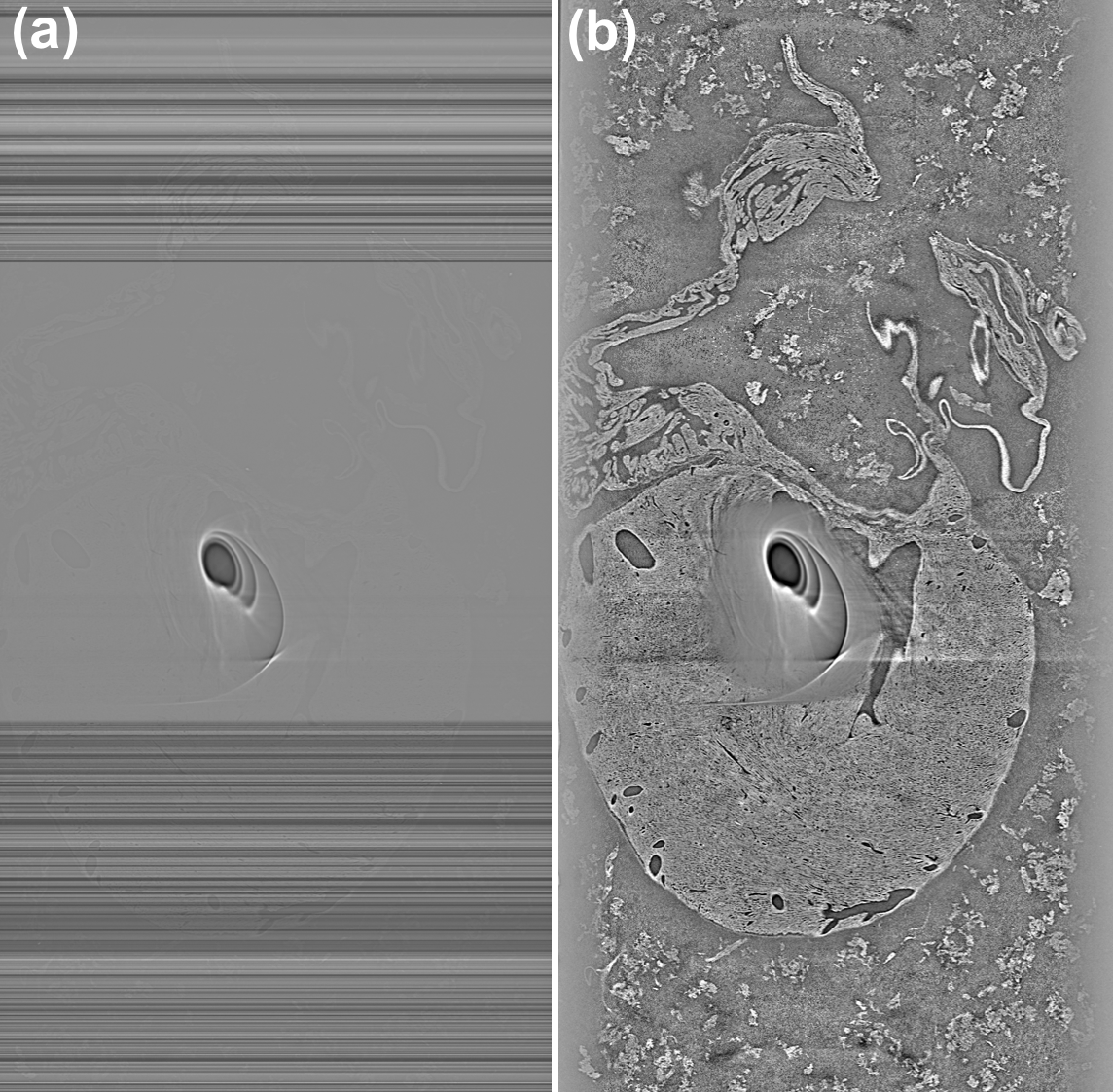

Supplement: S1 Fig — A longitudinal reslice of the image data set corresponding to sample IUGR4 (a) before and (b) after applying the Normalize Local Contrast filter where a lot of illumination artefacts can be clearly seen in the original dataset. (TIFF) [file pone.0218192.s001.tiff]

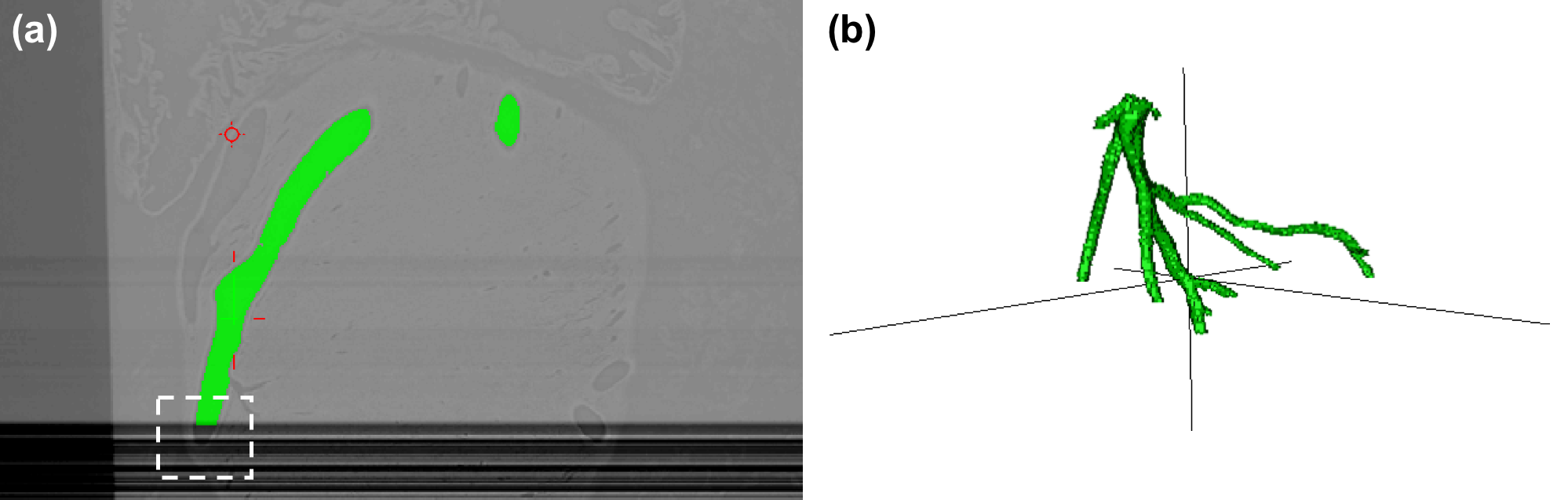

Supplement: S2 Fig — (a) Incomplete segmentation of the coronary arteries (in green) when illumination artefacts are present (dashed line white box). (b) Three-dimensional reconstruction of the partial segmentation of the coronary arterial tree. (TIFF) [file pone.0218192.s002.tiff]

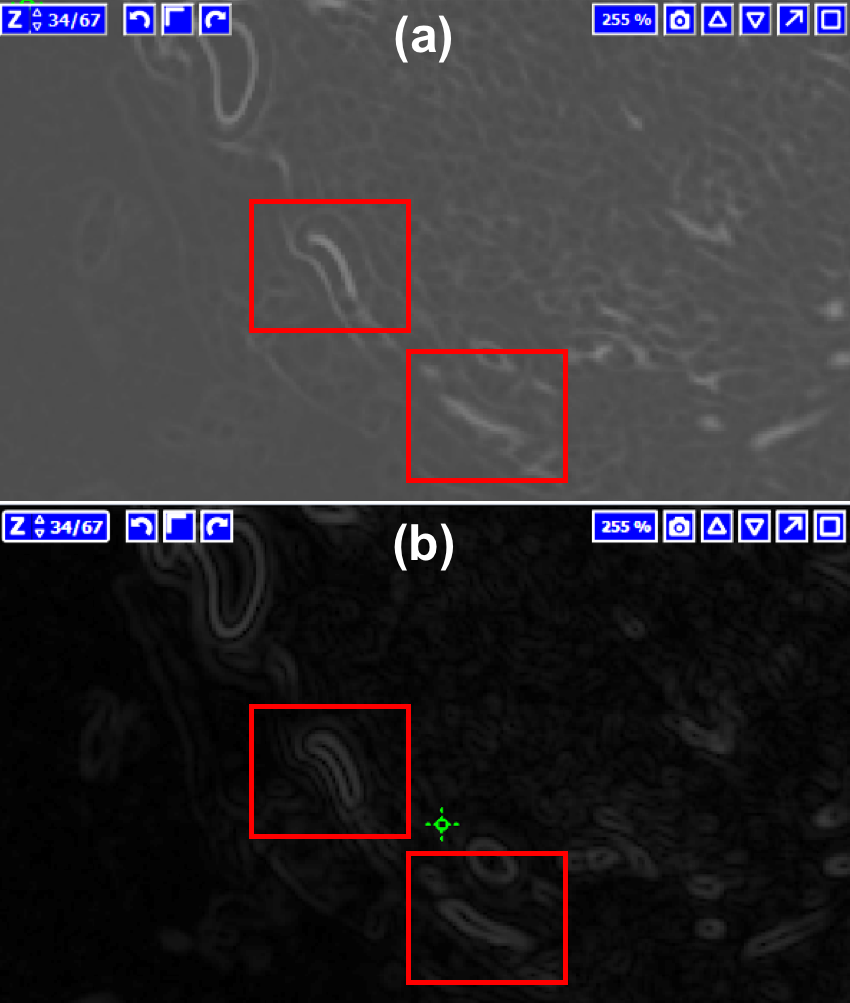

Supplement: S3 Fig — Results of the image filtering with (a) bright lines and (b) step edges filters to generate boundary maps in the same region. It can be clearly seen that the lumen of small vessels (red boxes) is only distinguishable in the step edge filtered image. (TIFF) [file pone.0218192.s003.tiff]

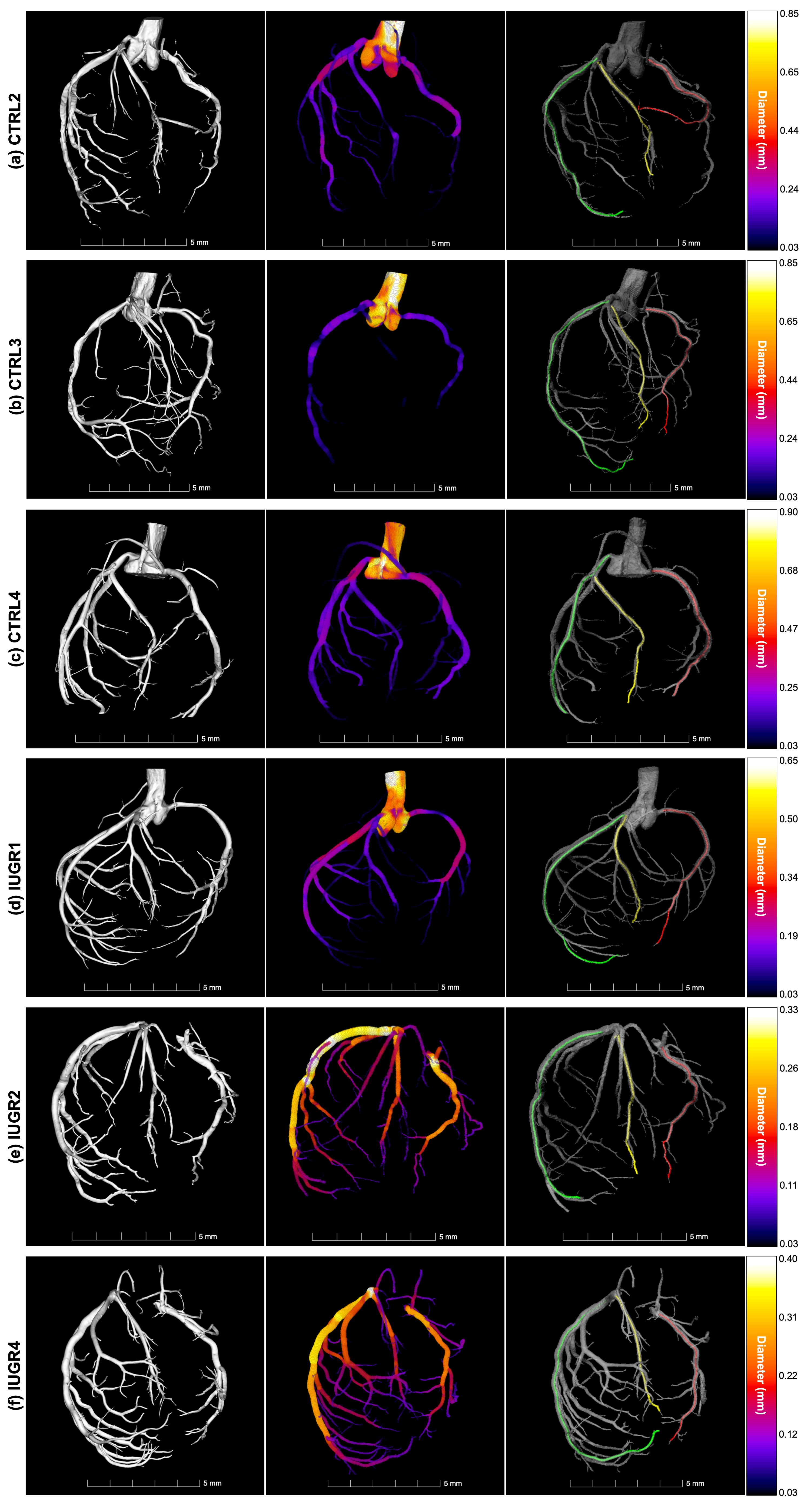

Supplement: S4 Fig — Three-dimensional representation of the segmented coronary tree (left panel) together with their corresponding colour visualisation of the estimated lumen diameter (middle panel) for the reaming 3 control (a-c) and 3 IUGR (d-f) fetal hearts. The right panel depicted the three main coronaries that were quantified for each dataset indicated in different colours: right coronary artery (RCA) in red, left coronary circumflex artery–including the left main stem (LCX) in green, and left anterior descending (LAD) artery in yellow. (TIFF) [file pone.0218192.s004.tiff]
