# Supplementary material for: Structural coronary artery remodelling in the rabbit fetus as a result of intrauterine growth restriction
Source: PLoS One. 2019 Jun 21;14(6):e0218192. doi: 10.1371/journal.pone.0218192 (PMC6588274; doi:10.1371/journal.pone.0218192)
Supplement: S1 File — Supplementary methods. (PDF) [file pone.0218192.s005.pdf]

Supporting methods for:
Structural coronary artery remodelling in the rabbit fetus as a result of intrauterine growth restriction

Patricia Garcia-Canadilla[1¶*], Tom de Vries[2¶], Anna Gonzalez-Tendero[3,4], Anne Bonnin[5,6], Eduard Gratacos[3,4,7,8], Fatima Crispi[3,4,7], Bart Bijnens[1,9] and Chong Zhang[1]

[1] Department of Information and Communication Technologies, Universitat Pompeu Fabra, Barcelona, Spain

[2] Medical Image Analysis, Technische Universiteit Eindhoven, Eindhoven, Netherlands

[3] BCNatal | Fetal Medicine Research Center (Hospital Clínic and Hospital Sant Joan de Déu), University of Barcelona, Barcelona, Spain

[4] Institut d'Investigacions Biomèdiques August Pi i Sunyer (IDIBAPS), Barcelona, Spain

[5] European Synchrotron Radiation Facility, Grenoble, France

[6] Paul Scherrer Institute, Villigen, Switzerland

[7] Centre for Biomedical Research on Rare Diseases (CIBER-ER), Madrid, Spain

[8] Institut de Recerca Sant Joan de Déu, Esplugues de Llobregat, Spain

[9] Institución Catalana de Investigación y Estudios Avanzados (ICREA) , Barcelona, Spain

***Corresponding author**

E-mail: patricia.garciac@upf.edu (PGC)

[¶] Both authors contributed equally to this work and therefore share the first authorship.

Image pre-processing

Five datasets had some illumination artefacts that can be visualised as bright and dark lines when doing a longitudinal reslice of the image dataset, as illustrated in Figure A in S1 Fig. These artefacts can lead to contrast differences between adjacent slices that difficult the subsequent automatic segmentation of the coronary arteries with *Ilastik* [1] as shown in S2 Fig. Therefore, in order to reduce the artefacts and homogenise the image contrast while keeping image quality, the build-in Fiji [2] function *Normalise Local Contrast*, which is part of the *Integral Image Filters* plugin, with a filter size of 40x40 pixels and a standard deviation of 3.0 was used. As can be seen in Figure B in S1 Fig the artefacts were completed removed after filtering.

Segmentation of the coronary arteries

To segment the coronary arterial tree, the semiautomatic workflow *Carving* of *Ilastik* was used. *Carving* uses a seeded watershed algorithm for iterative object carving from image data [3]. The seeded watershed algorithm relies on apparent object boundaries in the image data. To define a good object boundary map, the algorithm offers different filtering options depending of the object boundary: bright lines, dark lines and step edges. In our image datasets the vessel lumen is dark, and the vessel wall is brighter compared to the surrounding tissue. Therefore, two different filtering options were investigated: bright lines and step edges. Both bright lines and step edges filters give a good result for big vessels. However, on smaller vessels the result of bright lines filter was not good being difficult to distinguish the lumen inside the small vessels as illustrated in S3 Fig. Therefore, the step edge filter with a small filter size value was used to construct the boundary map. Some seeds for the object (vessel lumen) and background were manually provided. From these seeds a segmentation was automatically calculated. The segmentation could be refined interactively by providing additional seeds (see online video [4]).

References

1. Sommer C, Straehle C, Kothe U, Hamprecht FA. Ilastik: Interactive learning and segmentation toolkit. 2011 IEEE International Symposium on Biomedical Imaging: From Nano to Macro. IEEE; 2011. pp. 230–233. doi:10.1109/ISBI.2011.5872394
2. Schindelin J, Arganda-Carreras I, Frise E, Kaynig V, Longair M, Pietzsch T, et al. Fiji: an open-source platform for biological-image analysis. Nat Methods. 2012;9: 676–82. doi:10.1038/nmeth.2019
3. Sträehle CN, Köthe U, Knott G, Hamprecht FA. Carving: scalable interactive segmentation of neural volume electron microscopy images. Med Image Comput Comput Assist Interv. 2011;14: 653–60.
4. Vries T de. Ilastik Preprocessing and Segmentation - Tom de Vries [Internet]. 2018. Available: https://youtu.be/P6ZCa1Eog9c
